# Results of Coordinated Investigations of a National Colorectal Cancer Education Campaign in Appalachia

**Published:** 2006-03-15

**Authors:** Eugene J Lengerich, Angel Rubio, Evelyn A Knight, Stephen W Wyatt, Pamela K Brown

**Affiliations:** Pennsylvania State University, Health Evaluation Sciences; Dr Lengerich is also associated with the Penn State Cancer Institute and the Penn State College of Medicine; College of Public Health and Markey Cancer Center, University of Kentucky, Lexington, Ky; College of Public Health and Markey Cancer Center, University of Kentucky, Lexington, Ky; College of Public Health and Markey Cancer Center, University of Kentucky, Lexington, Ky; West Virginia University, Morgantown, WVa

Appalachia is largely rural, and residents have less contact with physicians, lower levels of preventive care, and less health insurance coverage for the nonelderly than the general U.S. population. The incidence of colorectal cancer in Appalachian areas of Kentucky, Pennsylvania, and West Virginia has been found to be greater than the incidence in other areas of the United States. To investigate community-based methods to address this health disparity, the Appalachia Cancer Network (ACN) developed and implemented three coordinated investigations of *Screen for Life: National Colorectal Cancer Action Campaign*, a national, multiyear, multimedia campaign to promote colorectal cancer education and screening among men and women aged 50 years and older. Together, these three investigations represent coordinated research from a network of investigators working with state and national partners in largely rural regions. The investigations provide important insights into the perceived effectiveness and methods for dissemination of *Screen for Life *messages in rural Appalachia.

## Colorectal Cancer and Appalachia

From 1994 through 1998, the incidence rates of colon cancer in the Appalachian counties of Kentucky, Pennsylvania, and West Virginia were 13% greater than the incidence rates from the Surveillance, Epidemiology, and End Results population of the United States, and incidence rates of rectal cancer in those counties were 19% greater (1). Incidence rates for each of the invasive cancer stages — local, regional, and distant — were generally higher as well. Appalachia also has been found to have a higher colorectal cancer mortality rate (2,3).

The incidence and mortality data suggest low use of colorectal cancer screening in rural Appalachia, because screening has been shown to reduce colorectal cancer incidence and mortality (4-8). Indeed, use of colorectal cancer screening has been found to be low in rural areas of the United States (9). Unfortunately, the prevalence of screening for colorectal cancer in Appalachia has not been reported previously. However, in 2002, fewer than half of adults aged 50 years and older in Kentucky (43.9%), Pennsylvania (48.0%), and West Virginia (40.4%) reported ever having had a sigmoidoscopy or colonoscopy; the nationwide estimate was 48.1% (10).

The level of awareness and knowledge of clinical guidelines and recommendations for regular screening for colorectal cancer among the general population in Appalachia is also unknown. However, because Appalachia is largely rural, cancer care is disconnected (11), meaning that residents of rural areas generally have less contact with physicians, lower levels of preventive care, less health insurance coverage for the nonelderly, and less access to clinical trials than the general U.S. population. Also contributing to less-than-optimal preventive and clinical care are systemic factors related to rural life, including longer distances to health care facilities and providers, lack of public transportation, few community services, and high rates of poverty and unemployment.

## The ACN and *Screen for Life*

Funded by the National Cancer Institute and centered in three academic institutions, the ACN brings new cancer prevention and control information to mobilized communities within Appalachian counties in eight states. From 1992 to 2000, more than 1800 community and professional leaders were mobilized to develop more than 40 community coalitions that have implemented at least 1000 local cancer control activities in 71 Applachian counties (12). The Markey Cancer Center at the University of Kentucky is the administrative core and directs ACN's Central Highlands region in Kentucky, Tennessee, and Virginia. Working with cooperative extension agents and the Penn State Cancer Institute, the Pennsylvania State University leads the Northern ACN in Pennsylvania and New York. Finally, the Mary Babb Randolph Cancer Center at West Virginia University directs the North Central ACN, including West Virginia, Ohio, and Maryland.

*Screen for Life* is a national, multiyear, multimedia campaign to educate men and women aged 50 years and older about the importance of having regular colorectal cancer screening tests (13). Launched in 1999, *Screen for Life* was designed, developed, and implemented by the Centers for Disease Control and Prevention (CDC) and the Centers for Medicare and Medicaid Services (formerly the Health Care Financing Administration), with technical support from the National Cancer Institute. *Screen for Life* campaign messages and materials, including brochures, posters, and public service announcements for radio and television (14), were developed after formative research, which included an extensive review of published communication and behavioral science literature and more than 100 focus groups of men and women aged 50 years and older conducted in more than 40 U.S. cities.

In 2002, ACN investigators began to consider the potential of *Screen for Life* to address the high colorectal cancer incidence in rural Appalachia (11). The following questions were raised:

- Were the state health departments in Appalachia disseminating *Screen for Life*? If so, what were the impressions of state leaders about *Screen for Life* and its materials?
- Were the *Screen for Life* print materials well received by the Appalachian population?
- Could community coalitions complement regional and statewide media efforts by effectively disseminating *Screen for Life* at the local level?

ACN developed three coordinated investigations to begin to address these questions, and their subsequent reports are included in this issue of *Preventing Chronic Disease*. Together, these three reports represent planned, coordinated research from a network of investigators working with state and national partners in a medically underserved region.

Vanderpool and Coyne (15) interviewed three state health department staff members, three ACN regional directors, and seven community-level intermediaries to examine the perceived effectiveness and state- and region-level dissemination of *Screen for Life* materials. They found that respondents reported *Screen for Life* materials to be generally effective in raising awareness of screening and that a formal evaluation of the effectiveness of *Screen for Life* materials among rural Appalachians was needed. Most respondents reported state and regional dissemination; dissemination through ACN may have been greater than dissemination through state agencies. However, respondents reported that competing priorities, a lack of funding, and absence of a plan of action for implementing the *Screen for Life* media campaign as part of a comprehensive colorectal cancer education campaign prevented more effective dissemination of* Screen for Life* materials.

Davis et al (16) conducted four focus groups with residents at least 50 years of age and three focus groups with physicians' office staff members in rural Appalachia to assess the perceived effectiveness of *Screen for Life* materials. They found that both groups preferred *Screen for Life* materials to other materials about colorectal cancer screening. Both groups also similarly ranked the importance of various concepts and facts related to colorectal cancer prevention among rural Appalachians. Although the *Screen for Life* materials were developed with limited input from the Appalachian population, and ways to improve the material were identified, the *Screen for Life* materials were generally preferred and considered by participants to effectively communicate information about colorectal cancer screening.

Ward et al (17) used a controlled, community intervention pilot study in 18 Appalachian counties to examine whether the involvement of rural community coalitions would increase the dissemination of *Screen for Life* materials. They found that the coalition arm recruited approximately three times more community organizations to disseminate *Screen for Life* materials than the noncoalition arm of the study. Representatives of community organizations reported that the presence of a coalition was an important factor in their decision to participate. This pilot study found support for the hypothesis that community coalitions may be an effective mechanism to increase the dissemination of colorectal cancer education material. In addition, the study found differences in the participation of organizations by organization type.

The three reports in this issue of *Preventing Chronic Disease* provide important insights into the perceived effectiveness and methods for dissemination of *Screen for Life* materials in rural Appalachia. Although these studies were not designed to demonstrate conclusively the utility and best mechanism for dissemination of *Screen for Life* in rural Appalachia, there appears to be substantial potential for the *Screen for Life* materials and campaign in rural Appalachia. Two observations are warranted. First, use of *Screen for Life* materials is probably limited at the local level in rural Appalachia. To begin to overcome these local shortcomings, state and regional cancer education campaigns should engage local individuals, health care practices, and organizations. Second, the impact of *Screen for Life* materials and the *Screen for Life* campaign on the prevalence of colorectal cancer screening in rural Appalachia has not been examined. However, it appears to be a reasonable hypothesis that *Screen for Life* materials disseminated at the state, regional, and community levels could increase the number of individuals in rural Appalachia who seek colorectal cancer screening. A multilevel approach to information dissemination may help ensure that *Screen for Life* messages reach rural residents, thereby increasing the possibility of a reduction in the colorectal cancer screening disparity for residents of rural areas.
